# Multifocal Langerhans' Cell Histiocytosis in an Adult

**DOI:** 10.22038/IJORL.2023.65286.3238

**Published:** 2023-07

**Authors:** Navid Nourizadeh, Mohammad Reza Majidi, Mohammad Reza Afzalzadeh, Shirin Taraz Jamshidi

**Affiliations:** 1 *Sinus and Surgical Endoscopic Research Center, Faculty of Medicine, Mashhad University of Medical Sciences, Mashhad, Iran.*; 2 *Department of Pathology , Faculty of Medicine ,Mashhad university of medical sciences , Mashhad ,Iran.*

**Keywords:** Langerhans’ Cell Histiocytosis, Mastoiditis, Neck mass

## Abstract

**Introduction:**

Multifocal Langerhans' cell histocytosis is a rare condition that can affect multiple organs and manifest in various scenarios. While the condition is more commonly found in children, it can also occur in adults.

**Case Report:**

A 43-year-old female presented with refractory otorrhea and had a rubbery neck mass in the left mid-cervical area, as well as an itchy eczematoid lesion in the left parietal area. The otic lesion was eventually resected, and histopathologic examination confirmed the diagnosis of Langerhans histiocytosis.

**Conclusions:**

Although rare in adults, Langerhans histiocytosis should be considered as one of the differential diagnoses for ear canal polyps. If diagnosed, medical treatment should be pursued.

## Introduction

Since 1987, the term hystiocytosis has been used to describe a group of diseases that were previously known as eosinophilic granuloma, Hand-Schüller-Christian disease, and Letterer-Siwe disease. These three conditions were first linked by American pathologist Louis Lichtenstein in 1953, who observed an abnormal accumulation of hystiocytes in the patients' tissues.

These hystiocytes were characterized by abnormal cytoplasmic substances, which led to the name hystiocytosis X due to unknown etiology. Today, these cells are known as langerhans cells, and their abnormal accumulation and enlargement is the hallmark of the disease's pathogenesis.Collectively, these conditions are referred to as LCH, which represents a spectrum of clinical severity of a major disorder. 

Eosinophilic granulomas are a benign form of the disease and are characterized by either single or multiple osteolytic lesions. Hand-Schüller-Christian disease is a chronic, multifaceted condition that involves several skin lesions and soft tissues, including the skin and mucous membranes.

In about 10% of cases, the classic "Christian triad" symptoms of insipid diabetes, bony lesions of the skull, and exophthalmus may present. Letterer-Siwe disease, on the other hand, is an acute and catastrophic illness marked by extensive skin eruptions, lung infiltration, and hepatosplenomegaly.

Histopathologically, all three conditions represent benign, acute, and chronic forms of a similar systemic disease process (1).

While LCH is rare in children, there have been few reports of auditory involvement in adults (2). In this case report, we present LCH as an educational example and reminder to consider it in the differential diagnosis of auditory canal polyps.

## Case Report

The patient is a 43-year-old white female who has been suffering from chronic left ear otorrhea for the past 20 months, despite undergoing various topical antibiotic treatments.

**Fig 1:**
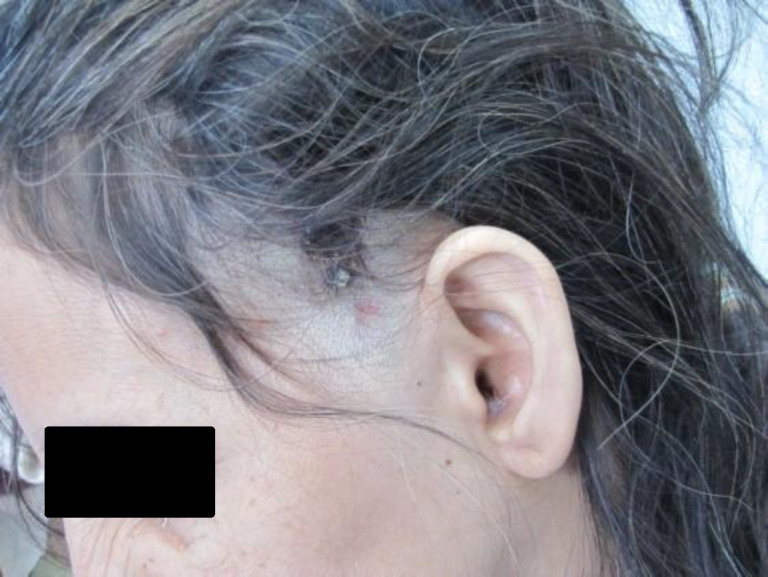
Left seborrhoic rash on left parietal region

**Fig 2:**
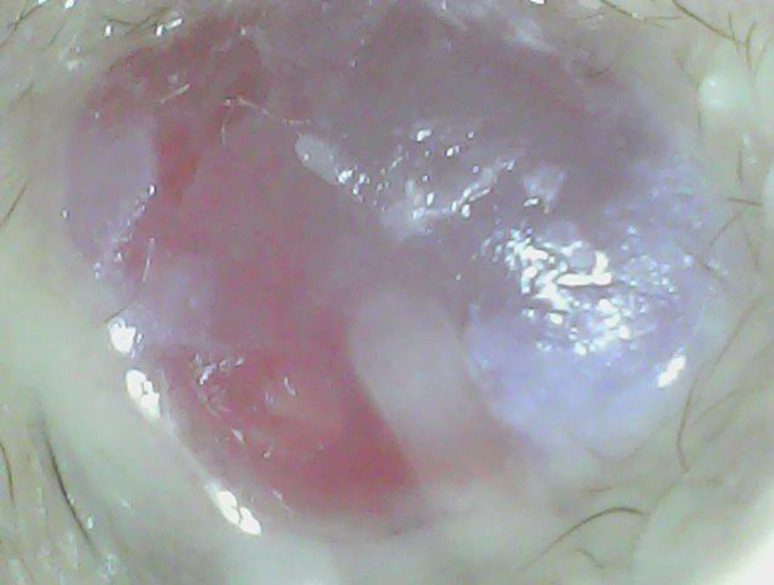
Occluded ear canal with polypoid tissue and obscured eardrum

**Fig 3:**
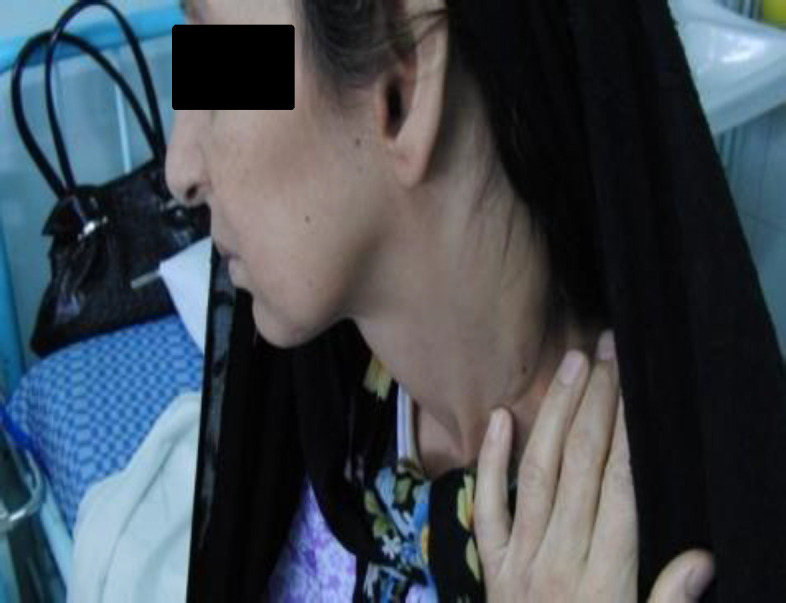
A left-sided mobile nontender 3*3 neck mass with a rubbery texture

Additionally, she developed a painless mass in her left neck six months ago and has been experiencing a seborrheic rash on her left parietal region (Figures 1-3).

During an otoscopic examination, it was observed that both ear canals were occluded with polypoid tissue, which also obscured the eardrum (Figure 2).

**Fig 4:**
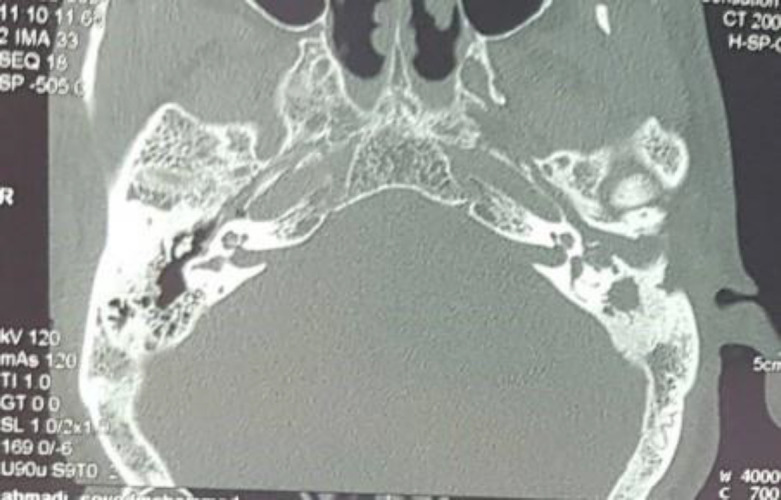
Coronal temporal CT scan shows sclerotic left mastoid due to chronic matoiditis and soft tissue density in mastoid middle ear and external auditary

However, there was no palpable tenderness in the mastoid areas. Audiometrical studies revealed that the tympanogram of both sides were type B, with moderate conductive hearing loss. A temporal CT scan was performed, which showed left mastoid sclerosis and soft tissue density in the mastoid middle and external ear, as shown in figure 4.

**Fig 5:**
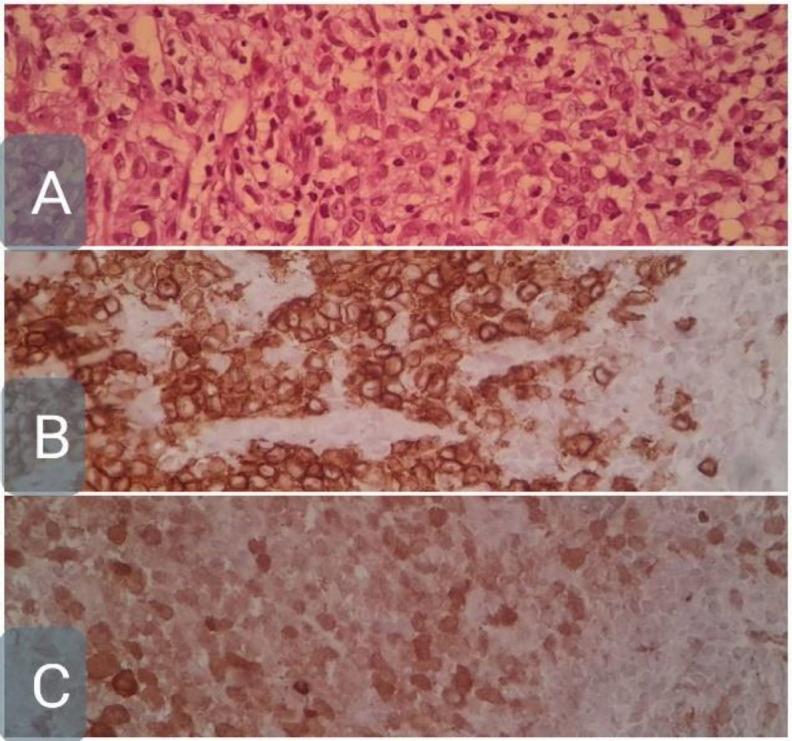
A. H&E (Hematoxylin and Eosin) staining of infiltrated histiocytes with round to oval nuclei having nuclear clefts and adequate cytoplasmic appearance, along with some eosinophils scattered throughout.B. Immunohistochemical staining with anti-CD1a antibody showing positive results.C. Immunohistochemical staining with S100 marker showing positive results

During mastoidectomy, a hypertrophic mastoid membrane was observed along with obvious destruction of the ossicles and external auditory canal. Pathologic studies revealed chronic inflammation and diffuse monocyte infiltration with oval-shaped cleaved nuclei and acidophilic cytoplasm with reaction to CD1a and S100 markers, confirming the diagnosis of Langerhans histiocytosis (Figure 5). A neck biopsy also confirmed the diagnosis.

As a result of these findings, the patient was referred to the oncology ward for further treatment approaches.

The medical oncology department initiated systemic treatment, which included a combination of prednisone and vinblastine. Following completion of the first session of chemotherapy, the patient returned to her home country.

Fortunately, the patient's response to the treatment was excellent. Within four weeks of starting induction therapy, noticeable improvements were observed.

Over the course of the next 3 months of induction therapy, the otologic manifestation continued to improve.At the end of the induction therapy, the patient underwent an examination revealing a normal tympanic membrane without any perforation or aural polyps.

## Discussion

LCH is a disease that predominantly affects children, with an average age of onset at three years. However, it can also occur in adults, typically in the range of 30 to 39 years of age (3). Temporal bone involvement is observed in approximately 14% to 61% of children with LCH, but limited cases have been reported in adults (2-4).

Otological manifestations of LCH include otorrhea, polyps of the outer canal, post-auricular swelling, conductive hearing loss, and rarely facial paralysis and vertigo (5).

Ear involvement is bilateral in 30% of cases, while in 25% of patients, the only symptom is ear disease (6).

Physicians should consider LCH in patients with refractory otorrhea and radiological evidence of soft tissue density in the middle and outer ear canal. A typical manifestation of LCH is a homogenous soft tissue lesion with sclerotic margins that enhance homogenously upon intravenous contrast injection (7).

Biopsy is the gold standard test for diagnosis, which reveals Langerhans cells along with infiltration of plasma cells, lymphocytes, and eosinophils through immunohistochemistry methods. In cases where LCH is suspected, frozen section biopsy during surgery can aid in the diagnosis and prevent the surgeon from resorting to radical surgery (8).The treatment of LCH depends largely on the pattern of the disease. Localized LCH is treated with surgery and steroid injections into the lesion, while immunosuppression is recommended for systemic LCH. Chemotherapy with vinblastine and steroids is also used as a treatment option for systemic LCH (9). Most patients with an ear problem have a systemic form of LCH, so surgical treatment is recommended only for diagnosis purposes. It should be noted that surgical treatment cannot completely eliminate the lesion, and complications such as facial nerve palsy, postoperative fistula, and deafness may occur thereafter. Therefore, surgical interventions are considered high-risk procedures in LCH patients with otological manifestations and are avoided when possible (10). If ear surgery is performed, close surveillance is crucial in ensuring that disease recurrence following initial treatment is detected early. As such, it is recommended that patients undergo surveillance imaging with MRI and/or PET every 6 months since up to 50% of patients may experience recurrence. 

These imaging modalities allow for the detection of residual lesions or new lesions, which can be treated promptly to prevent further complications. Additionally, regular follow-up visits with an oncologist and an otolaryngologist are necessary to monitor patients' overall health and ensure that they are responding well to treatment. Early detection of recurrent LCH is critical in preventing the progression of the disease and reducing the risk of permanent damage to affected organs (11).

## Conclusion

Although LCH usually occurs in children, it should be taken into account in any adult patient who have resistant otorrhea or mastoiditis. If diagnosed, treatment should be done medically.
